# Schuylkill Valley Veterinary Medical Association

**Published:** 1896-10

**Authors:** Otto Noack

**Affiliations:** Corresponding Secretary


### SOCIETY PROCEEDINGS.

### SCHUYLKILL VALLEY VETERINARY MEDICAL ASSOCIATION.

THE semi-annual meeting of the Association was held at City Hall, Reading, September 7, 1896. Through late arrival of the members the meeting convened at 1 o'clock P.M., with Dr. Burkholder in the chair. To roll-call the following members responded: Drs. Sallade, Burkholder, Fridirici, McCarthy, Noack, Faughnan, Kershner, Brackbill. Visitor: Dr. Dreibelbis, from Dreibelbis Station. Reading of the minutes of the previous meeting followed. The Recording Secretary was directed to add to the minutes that the Association had become an association of graduates, and that there was adopted an initiation fee of $3, after which the minutes were approved. Secretary Fridirici had to leave on account of illness, and Dr. Noack acted as Secretary.

Dr. F. H. Snyder, from Ashland, a graduate from the National College, Class of 1896, was proposed for membership. The trustees, Drs. Sallade, Kershner, and Dr. Brackbill, who was appointed in the absence of Dr. Bieber, reported favorably, and Dr. Snyder was duly elected to membership.

Under new business Dr. Noack reported that a board of health was to be installed in Adamstown, and moved that some action to place thereon a veterinarian should be taken by the society. This motion was sustained by Drs. McCarthy and Faughnan, and the Association directed the Corresponding Secretary to act in this matter and correspond with the council of that borough.

A communication of the committee of the Cosmos Club, for the erection of a Pasteur monument, asking for contributions, was read, and the Secretary was instructed to communicate with all the members and report at the next meeting.

After approving bills, etc., the reading of papers was in order. Dr. Sallade read his well-prepared paper, "Social Position of the Veterinarian," and the society tendered its thanks to the writer for his able handling of the subject. Dr. Faughnan followed with his paper, "Tetanus," which was thoroughly discussed by all the members present. Papers which will be read at the next meeting, to be held at Hotel Woll, Pottsville, December 16, 1896, are: "Sporadic Pneumonia," by Dr. Potteiger; "Shoeing," by Dr. Yingst; "Laminitis," by Dr. McCarthy; and "Infectious Pneumonia," by Dr. Brackbill. The meeting adjourned at 6 P.M.

OTTO NOACK,
Corresponding Secretary.

